# Characterization and Identification of a New Daidzein Reductase Involved in (*S*)-Equol Biosynthesis in *Clostridium* sp. ZJ6

**DOI:** 10.3389/fmicb.2022.901745

**Published:** 2022-05-20

**Authors:** Yunfei Hu, Chunfang Yang, Can Song, Weixuan Zhong, Baiyuan Li, Linyan Cao, Huahai Chen, Changhui Zhao, Yeshi Yin

**Affiliations:** Key Laboratory of Comprehensive Utilization of Advantage Plants Resources in Hunan South, College of Chemistry and Bioengineering, Hunan University of Science and Engineering, Yongzhou, China

**Keywords:** (*S*)-equol, gut microbes, *Clostridium*, functional gene, daidzein reductase, dihydrodaidzein, oxidase

## Abstract

(*S*)-equol (EQ) is an isoflavone with high estrogen-like activity in the human body, and is only produced by some gut bacteria *in vivo*. It plays an important role in maintaining individual health, however, the dearth of resources associated with (*S*)-EQ-producing bacteria has seriously restricted the production and application of (*S*)-EQ. We report here a new functional gene *KEC48*-07020 (K-07020) that was identified from a chick (*S*)-EQ-producing bacterium (*Clostridium* sp. ZJ6, ZJ6). We found that recombinant protein of K-07020 possessed similar function to daidzein reductase (DZNR), which can convert daidzein (DZN) into *R/S*-dihydrodaidzein (*R/S*-DHD). Interestingly, K-07020 can reversely convert (*R/S*)-DHD (DHD oxidase) into DZN even without cofactors under aerobic conditions. Additionally, high concentrations of (*S*)-EQ can directly promote DHD oxidase but inhibit DZNR activity. Molecular docking and site-directed mutagenesis revealed that the amino acid > Arg75 was the active site of DHD oxidase. Subsequently, an engineered *E. coli* strain based on K-07020 was constructed and showed higher yield of (*S*)-EQ than the engineered bacteria from our previous work. Metagenomics analysis and PCR detection surprisingly revealed that K-07020 and related bacteria may be prevalent in the gut of humans and animals. Overall, a new DZNR from ZJ6 was found and identified in this study, and its bidirectional enzyme activities and wide distribution in the gut of humans and animals provide alternative strategies for revealing the individual regulatory mechanisms of (*S*)-EQ-producing bacteria.

## Introduction

In recent years, several long-term follow-up large-scale prospective studies have revealed that eating habits with high isoflavone content from soy products demonstrate an inverse association with cerebrovascular diseases, climacteric period syndrome, and risk of death, in which (*S*)-equol (EQ) may play an important role (Zheng et al., 2019; Battaglia et al., 2020; Katagiri et al., 2020; Ma et al., 2020; Matsumoto et al., 2020). As a soy isoflavone it possesses high estrogen-like activity, (*S*)-EQ, rather than (*R*)-EQ, can be an excellent alternative for estrogen in the human body (Setchell and Clerici, 2010). Individuals who are capable of producing (*S*)-EQ—as opposed to those who are not—have lower triglyceride and high-density lipoprotein plasma levels and show lower risk rates for cardiovascular diseases (Ahuja et al., 2017; Yoshikata et al., 2017). For menopausal women, (*S*)-EQ producers are at a lower risk of developing osteoporosis (Lambert et al., 2017; Mayo et al., 2019). Not only that, (*S*)-EQ is known to possess several biological activities, such as anti-oxidative stress, anti-inflammatory, and antibacterial activities (Li, 2019; Mayo et al., 2019; Tanaka et al., 2019), and it can be used for the treatment of atherosclerotic lesions, major symptoms among postmenopausal women (Chen et al., 2019; Yoshikata et al., 2021) and estrogen-related cancer (Daily et al., 2019; Kwon et al., 2019).

(*S*)-EQ is not a natural component of soybeans and also cannot be synthesized by the human body. (*S*)-EQ is one of the final products of daidzein (DZN) metabolism that occurs in enteric bacteria. Larger differences in EQ producers vary between populations, of which there are 30%–60% in Asia, but only about 25% in Western countries (Setchell and Cole, 2006). In light of recent findings, the presence of (*S*)-EQ-producing bacteria is a *sine qua non for* EQ producers. (*S*)-EQ is in great demand for research and biopharmaceuticals, and several studies have focused on (*S*)-EQ production in the context of synthetic biology.

**FIGURE 1:**
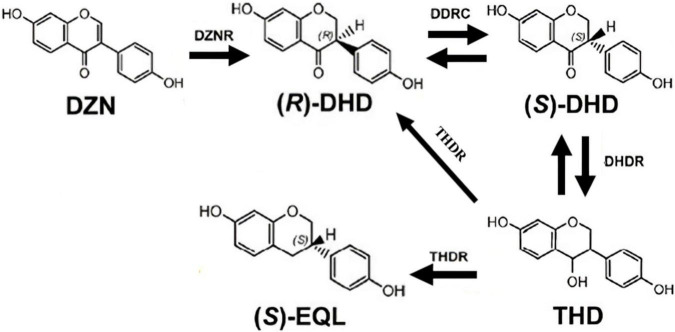
Model of the (*S*)-EQ biosynthetic pathway. It has been reported that at least four genes were involved in producing (*S*)-EQ, including daidzein reductase (DZNR), dihydrodaidzein racemase (DDRC), dihydrodaidzein reductase (DHDR), and tetrahydrodaidzein racemase (THDR). (*S*)-EQ production requires that daidzein is first metabolized into dihydrodaidzein by DZNR and DDRC, and further metabolized into tetrahydrodaidzein and then into (*S*)-EQ by DHDR and THDR.

Depending on study of the five known (*S*)-EQ-producing bacteria, *Lactococcus* sp. 20_92 (*Lac* 20_92) (Shimada et al., 2012), *Slackia isoflavoniconvertens* sp. HE8 (HE8) (Schroder et al., 2013), *Slackia* sp. NATTS (NATTS) (Tsuji et al., 2012), *Eggerthella* sp. YY7918 (YY7918) (Kawada et al., 2016) and *Adlercreutzia equolifaciens* sp. DSM19450^T^ (DSM19450^T^) (Florez et al., 2019), at least four genes have been found to be involved in producing (*S*)-EQ, including daidzein reductase (DZNR), dihydrodaidzein racemase (DDRC), dihydrodaidzein reductase (DHDR) and tetrahydrodaidzein racemase (THDR). (*S*)-EQ production in these bacteria requires that daidzein is first metabolized into dihydrodaidzein by DZNR, and further metabolized into tetrahydrodaidzein and then into (*S*)-EQ by DHDR and THDR (Figure 1). Based on the four functional genes, we and several other groups have constructed an *Escherichia coli* strain (DDDT, recombinant), which can convert DZN to (*S*)-EQ *in vitro*. However, the improvement of EQ production has not yet been explored in the field (Sajid et al., 2021).

In contrast to this, (*S*)-EQ-producing bacteria can also be detected in EQ non-producers, unfortunately, meaning it is difficult to improve the ability to produce EQ, even when isoflavone diet supplements were provided (Vazquez et al., 2017a; Iino et al., 2019; Yoshikata et al., 2019). A series of studies revealed that the ability of (*S*)-EQ production may be associated with EQ-related microbiota and intestinal environment (Vazquez et al., 2017b; Iino et al., 2019). To date, a detailed association between (*S*)-EQ-producing bacteria and the ability of individuals to produce (*S*)-EQ remain poorly understood. Thus, a key breakthrough for understanding individual differences in (*S*)-EQ producers have focused on specific bacteria, while detailed metabolic mechanisms are still being uncovered. In this study, a chick gut strain ZJ6 was isolated and identified as an (*S*)-EQ-producing bacterium, and a new functional gene involved in DZN metabolism was discussed.

## Materials and Methods

### Bacterial Culture and (*S*)-EQ-Producing Fermentation

*Clostridium* sp. ZJ6 (ZJ6) was isolated from the caecum of 15-day-old chicks and cultured for 24 h in Reinforced Clostridium Medium (RCM) under anaerobic conditions (10% H_2_, 10% CO_2_, and 80% N_2_) at 37°C. Next, these cells were sub-cultured 1:10 in fresh RCM with 40 μM DZN and dihydrodaidzein (DHD) for 24 h. The fermentation products of C1 were then subjected to analysis by HPLC. First, 1 mL of the fermentation supernatant was mixed with 0.7 mL of ethyl acetate by vigorous shaking and left to sit for 5 min, centrifuged for 5 min at 5,000 × *g*, and the supernatant was transferred to a new centrifuge tube. Each sample was extracted twice following the same procedure. Subsequently, all collected supernatants were dried by vacuum centrifugation and then dissolved in 0.2 ml of methanol. Finally, a Shimadzu HPLC CBM-20A system (Kyoto, Japan) with UV detector SPD-20A was used for HPLC analysis, and separations were performed on a SunFireTM C18 (5 μm, 4.6 × 250 mm) column. Elution was performed using a 0.1% trifluoroacetic acid aqueous solution (A) as well as methanol acetonitrile solution (B, 4:6, V/V), at a flow rate of 0.8 mL/min and a solution B gradient of 50% for 15 min. The detection wavelengths were 205 and 254 nm, the detection temperature was 30°C, and the injection volume was 5 μL.

### Screening for (*S*)-EQ-Producing Functional Genes

ZJ6 was grown in 100 ml of Gifu anaerobic medium (GAM) for 24 h, and after centrifugation at 5,000 × *g* for 5 min, bacteria were collected and genomic DNA was extracted using a Gram-positive bacterial DNA extraction kit (Qiagen, Hilden, Germany). Third generation whole genome sequencing was performed using a gridion sequencer (Nextomics, Wuhan, China). A whole-genome reference for ZJ6 was compared with reported DZNRs (*Lac* 20_92, Accession number AB558141.1; DSM19450, Accession number AP013105.1; HE8, Accession number JQ358709.1; NATTS, Accession number AB646272.1; YY7918, Accession number AP012211.1) using Local BLAST^1^ analysis. Potential functional genes were searched against the NCBI conserved domain database (CDD^2^), then the obtained (*S*)-EQ-producing functional gene clusters were analyzed using Motif Scan^3^. Likewise, other new functional genes were screened using the same method for reported DDRCs, DHDRs, and THDRs.

### Reductase Activity Detection of K-07020 *in vitro*

The *KEC48*-07020 (K-07020, GenBank accession QUN14249.1) gene was cloned into the plasmid pETDuet-1 (Novagen, Darmstadt, Germany) and then transformed into *E. coli* BL21 (DE3) (forward primer: 5′-cccgcggccgcatgaaaaacaaatattaccctca-3′, reverse primer: 5′- ccgcctaggttagatttgtctggctgctat-3′, restriction enzyme sites are underlined). After induction and expression in LB liquid medium, K-07020 protein was purified by Ni^2+^ affinity chromatography, and protein purity and concentration were determined by SDS-PAGE and BCA protein assay (Solebro, Beijing, China), respectively. The DZNR activity of purified K-07020 was detected in 1 mL of potassium phosphate buffer (100 mM, pH 7.0) under anaerobic conditions. Three micromoles K-07020 recombinant protease, 80 μM DZN, 1 mM NADPH or NADH, 5 mM sodium bisulfite, 2 mM dithiothreitol, and 1 mM phenylmethylsulfonyl fluoride were used. Following incubation at 37°C for 4 h, samples were extracted by ethyl acetate, and then used for HPLC detection. Elution was performed under 30% acetonitrile with a 0.1% acetic acid solution and a flow rate of 1 mL/min for 15 min. The detection wavelengths were 245 and 275 nm, the detection temperature was 30°C, and the injection volume was 10 μL. Moreover, the S-enantiomer and R-enantiomer of DHD were detected using a chiral detection procedure: separations were performed on a CHIRALCEL^®^ OJ3RCD-VB017 (0.46 cm I.D. × 15 cm L × 3 μm) column. Elution was performed using a 100% methanol, at a flow rate of 0.8 mL/min for 12 min. The detection wavelength was 254 nm, the detection temperature was 35°C, and the injection volume was 10 μL (sample was dissolved in methanol). To further characterize the role of EQ-production, K-07020 was constructed in an (*S*)-EQ producing engineered *E. coli* DDDT established in our preliminary work (Li et al., 2018), but the DZNR gene was replaced with K-07020 (K-07020DDT). Fermented productions were compared between DDDT and K-07020DDT, and *E. coli* BL21 (DE3) was used as a control.

### Oxidase Activity Assay of K-07020

To identify the new oxidase function of K-07020, the DZNRs from *Lac* 20_92 (GenBank: BAJ22678) and NATTS (GenBank: BAL46930) were selected as controls. Both the gene sequences of DZNRs were synthesized (Genscript, Shanghai, China) and cloned in pETDuet-1 and then transformed into *E. coli* using a similar method as with K-07020. pETDuet-1 *Lac* 20_92 DZNR BL21 (DE3) and pETDuet-1 NATTS-DZNR BL21 (DE3) were cultured in LB medium; then each of the recombinant proteins was induced and purified. Subsequently, recombinant proteins of K-07020, *Lac* 20_92 DZNR and NATTS DZNR were used to identify DHD oxidase function in 1 mL of 0.1 M PBS (pH7.0) under aerobic conditions. Three micromoles recombinant proteins, 80 μM of (*R*)- or (*S*)-DHD were used. Following incubation at 37°C for 4 h, samples were detected by the same method of reductase activity detection as mentioned previously.

Dihydrodaidzein oxidase activity using K-07020 recombinant protein was next detected *in vitro*. First, the optimal pH was determined in 1 mL 0.1 M citrate buffer (pH 5.0, 5.5, 6.0, or 6.5) or 0.1 M PBS (pH 7.0, 7.5, or 8.0) with 80 μM (*S*)-DHD and 3 μM recombinant protein from K-07020. Samples were incubated at 37°C for 2 h and then DZN was extracted for detection by HPLC. The temperatures for detection were 30, 34, 37, 41, and 45°C. After that, enzyme reactions were carried out for an hour. To determine the oxygen sensitivity, 3 mM cysteine hydrochloride was added according to optimal conditions above and used for enzyme activity detection under both aerobic and anaerobic conditions. To evaluate the effects of (*S*)-EQ (Daicel, Shanghai, China) and estradiol (Solebro, Beijing, China) on the reductase and oxidase activities of K-07020, 0.08, 0.16, 0.32, 0.64, 1.28, 2.56, and 4 mM of these compounds were added when performing enzyme reaction experiments.

### Molecular Docking Study

Molecular docking was used to investigate the key binding mode between the compound and K-07020 using Autodock vina 1.1.2 (Trott and Olson, 2010). The three-dimensional (3D) structure of K-07020 was built using SWISS-MODEL, a fully automated protein structure homology-modeling server. The 2D structure of the compound was drawn using ChemBioDraw Ultra 14.0 and converted to a 3D structure using ChemBio3D Ultra 14.0 software. The AutoDock Tools 1.5.6 package (Goodsell et al., 2021) was employed to generate docking input files. The ligand was prepared for docking by merging non-polar hydrogen atoms and defining rotatable bonds. The search grid for the K–07020 site for DHD was identified as center_x: –18.109, center_y: –4.869, and center_z: 6.716 with dimensions size_x: 21.75, size_y: 15, and size_z: 15. The search grid of the K-07020 site for DZN was identified as center_x: –18.109, center_y: –4.869, and center_z: 6.716 with dimensions size_x: 21.75, size_y: 15, and size_z: 15. The search grid of the K-07020 site for NADH was identified as center_x: –27.531, center_y: –8.022, and center_z: --1.784 with dimensions size_x: 15, size_y: 15, and size_z: 15.75. In order to increase the docking accuracy, the value of exhaustiveness was set to 20. For Vina docking, the default parameters were used if not otherwise mentioned. The best-scoring pose as judged by the Vina docking score was chosen and visually analyzed using PyMoL 1.7.6 software^4^. Meanwhile, SMART tool was used for function prediction^5^.

### Site-Directed Mutagenesis

Based on the molecular docking prediction, seven potential enzyme functional regions in K–07020 were selected for multiple amino acid mutation. The selective mutations were performed according to the polarity and charge of amino acids. For example, the positively charged amino acid R was mutated to a polar uncharged amino acid T, and the non-polar amino acid A was mutated to polar uncharged amino acid T (Supplementary Table 1). Briefly, two overlapping fragments for each mutation were amplified using fp1–rp1 and fp2–rp2, and overlap extension PCR with fp1–rp2 was performed for fusing full-length gene of mutants. The obtained mutants were cloned into pETDuet-1 using a one-step PCR kit (ent Vazyme, Guangzhou, China). Subsequently, recombinant plasmids were transformed into *E. coli* BL21 (DE3). Positive transformers were identified by PCR and then confirmed by Sanger sequencing (Sangon, Shanghai, China). Recombinant proteins from mutants were induced as small scale clones of K–07020, and enzyme function was determined from 1-mL whole-cell biocatalyst with 80 μM of DZN or DHD added, followed by incubation at 37°C for 4 h, and wild K-07020 was used as a control. Samples were then extracted and assessed by HPLC. Target mutants were selected for purification and used for quantitative detection with the same method as our activity assays for K-07020.

Following the results of multiple amino acid mutation, 16-point mutations were selected and used for identifying the key amino acid active site (Supplementary Table 2), and each point mutant was analyzed by using the above method. For further evaluating the key functional region of DHD oxidase activity, another site-directed mutagenesis screen was performed on the DZNR from *Lac* 20_92. The DZNR gene was used as a target, and amino acids at positions 72–78 were replaced by K–07020 with the key amino acid site of DHD oxidase activity (Supplementary Table 3), and DZNR was used as a control.

### Metagenomics Analysis of K-07020 in the Human Gut

To assess the distribution of these DZNR genes in the human intestine, metagenomic analysis was applied. Briefly, 4644 reference genomes were downloaded from the NCBI database of the human gut microbiome (Almeida et al., 2021) to test for similarity with K-07020 protein sequence using the tblastn tool, and >40% matching similarity and >90% coverage were used as the selective parameters. Second, based on the obtained reference genomes with high similarity and coverage greater than 80%, genes with the same types as ZJ6 were screened and downloaded from NCBI and tested against the K-07020 protein sequence using the above method. Afterward, all genes obtained from our two-step metagenomic analysis, as well as the seven reported DZNR genes (*Lac* 20_92: BAJ22678, YY7918: WP_013979957, HE8: WP_123220034, NATTS: BAL46930, DSM19450: WP_022741749, AUH-JLC159: AIC80887, Slackia: WP_123208882), were aligned using Evolutionary Genetic Analysis (MEGA) (Tamura et al., 2021).

One gene (00242_GENOMEO01471_13) with about 80% similarity and two genes (GUT_GENOME103990_60, GUT_GENOME127776_11) with about 40% similarity for K-07020 were synthesized. The enzyme activity of these genes was detected to be similar with K-07020.

### Analysis of Sequence Diversity

Based on the results of our metagenomic analysis, primers for cluster 1 and cluster 2 of these DZNRs were designed for PCR detection from the feces of humans and mice (Table 1). Briefly, 30 fecal samples were collected from 6–8-week-old female mice (Hunan SJA laboratory animal Co., Ltd., Changsha, China), and another 20 fecal samples were collected from 20–30-year-old healthy female volunteers. About 0.2–0.3 g of fecal samples were resuspended in water. The supernatants were obtained by centrifugation at 5,000 *g* for 10 min, which were then used for (*S*)-EQ production. The bacterial pellet was used for bacterial genomic DNA extraction (QIAGEN fecal DNA extraction kit, Hilden, Germany). PCR was performed to investigate the distribution of DZNRs, *E. coli* BL21 selected as a negative control.

**TABLE 1:** Primers used for detection of K-07020 and DZNRs.

Primer	Nucleotide sequence
Cluster 1 fp1	ATT(C)ACTTT(A)TGAA(G)CCATGGTATGAA
Cluster 1 rp1	CCACCATACATATCAGTACG
Cluster 2 fp1	GGCATC(T)GTG(T)TTC(T)ATGGAC(T)AAC
Cluster 2 rp1	TGTTG(A)TTGCGC(A)GGC(A)GAG(A/C)AGGAAG(A)TTG
Cluster 2 fp2	CAAC(T)TTCCTC(T/G)TCG(T)CCG(T)CGCAAC(T)AACA
Cluster 2 rp2	CGCAACCGATGCAG(A)GGC(G)CGG(A)ATG(A)TC

*Primers for cluster 1 were used to amplify K-07020-like genes, primers for cluster 2 were used to amplify the known DZNR-like genes, and due to the low sequence similarity of these genes, two pair of primers were designed; bases in brackets refer to degenerate base primers.*

### Statistical Analysis

Each treatment or group was assessed at least three times in parallel, and all data were statistically analyzed using IBM SPSS 20.0. The results for all data are displayed as the mean and standard deviation (SD). Statistical significance was determined using an independent sample *t*-test or Tamhane’s T2 (M) included in one-way ANOVA. *P* < 0.05 was considered to be statistically significant. Oxidase activity of K-07020 was determined according to the Michaelis–Menten Kinetics.

## Results

### (*S*)-EQ-Production and Functional Genetic Screening

**FIGURE 2:**
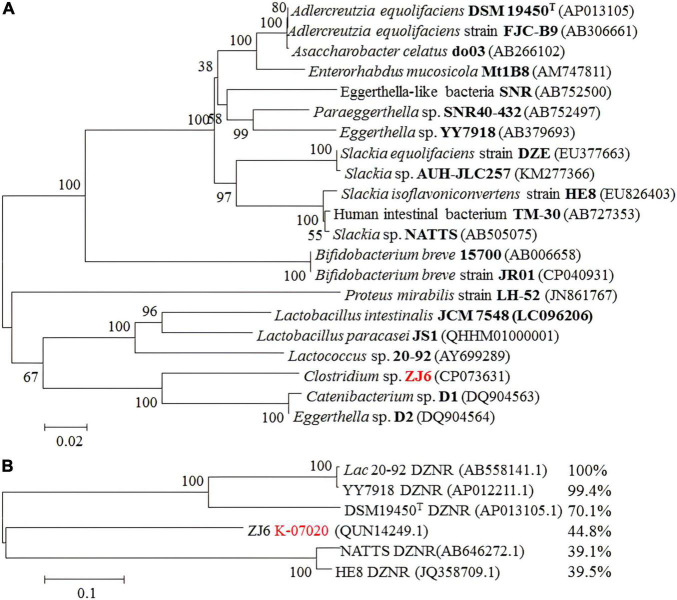
Phylogenetic tree of *Clostridium* sp. ZJ6 reveals relationship with equol-producing bacteria. The accession number is shown in parenthesis. Numbers at branch points indicated bootstrap values. The scale bar corresponds to 0.01/0.02 substitutions per nucleotide position. The name of *Clostridium* sp. ZJ6 in Genebank is *Clostridium* sp. C1. **(A)** Phylogenetic tree analysis of 16S rRNA gene for the main equol-producing bacteria. **(B)** Sequence identity analysis between K-07020 and reported DZNRs, where K-07020 had 44.8% sequence identity with *Lac* 20_92 DZNR.

ZJ6, which is a Gram-positive, filamentous, anaerobe bacterium isolated from chick cecum, can transform DZN into DHD and (*S*)-EQ under strictly anaerobic conditions (Supplementary Figure 1). However, when DHD instead of DZN was used as substrate, DZN was also detected in the fermentation broth of ZJ6. The complete genomic sequence of ZJ6 was next obtained (GenBank CP073631). Phylogenetic tree analysis of the 16S rRNA gene revealed that ZJ6 belonged to the genus *Clostridium* (Supplementary Figure 2). Compared to the main equol-producing bacteria, ZJ6 was more similar to *Catenibacterium* sp. D1 and *Eggerthella* sp. D2 (Figure 2A). After that, the genome of ZJ6 was used to compare sequence similarity and functional domains with the five reported DZNRs. A potential target gene in K-07020, which has 44.8% sequence identity with the DZNR from *Lac* 20_92, was found and selected for enzyme functional assays *in vitro* (Figure 2B).

### Enzyme Activity Analysis of K-07020

**FIGURE 3:**
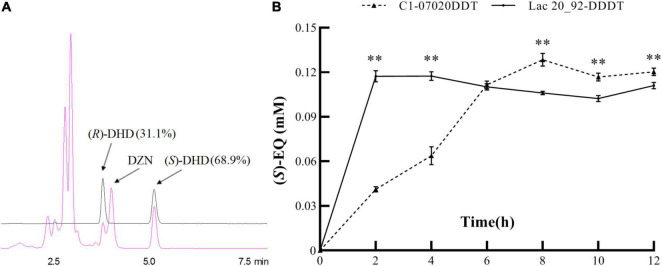
Reductase enzyme activity assays using K-07020. **(A)** Chiral detection of DHD by HPLC. Standard samples of (*R*)- and (*S*)-DHD were marked with a black line, and the fermentation sample was marked with a red line; K-07020 recombinant protein can transform DZN to both (*S*)-DHD and (*R*)-DHD. **(B)** Comparative analysis of (S)-equol production between K-07020DDT and *Lac* 20_92-DDDT. ** Represents the *P*-value less than 0.01 at the same time point.

K-07020 was cloned into pETDuet-1 and expressed in *E. coli* BL21 (DE3), and soluble recombinant proteins were purified and used for DZN transformation assays. The results showed that recombinant protein from K-07020 possessed DZNR activity that could transform DZN to DHD, and DZNR activity of K-07020 was dependent on NADH and anaerobic conditions (Supplementary Figures 3, 4). Interestingly, both (*S*)-DHD and (*R*)-DHD were detected by chiral HPLC analysis, and 68.9% of the total product was (*S*)-DHD (Figure 3A). To further evaluate the impact of K-07020 in (*S*)-EQ-producing, *Lac* 20_92 DZNR from the strain DDDT was replaced with K-07020 and used to construct K-07020DDT. When 0.15-mM DZN was used for fermentation, the production per unit time for DDDT (58.63 μM/h) was higher than K-07020DDT (16.05 μM/h). However, the fermentation results showed that K-07020DDT could produce (*S*)-EQ at about 10% higher levels than DDDT after fermentation for 8 h (Figure 3B).

**FIGURE 4:**
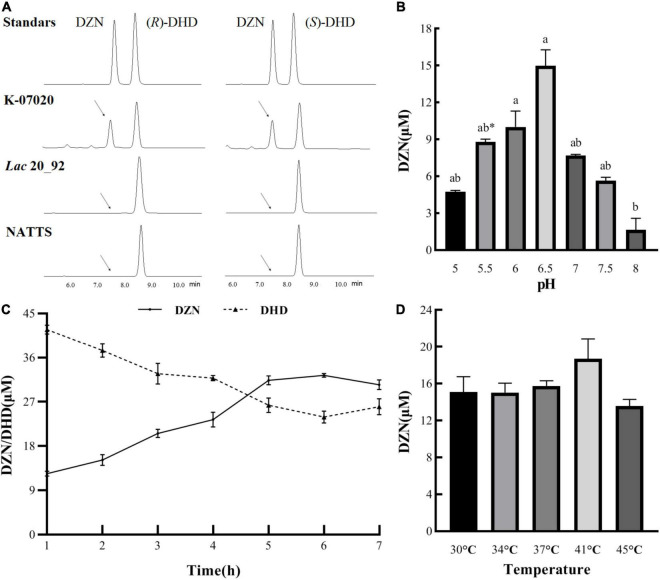
DHD oxidase activity with K-07020. **(A)** Detection the DHD oxidase activity of K-07020 under aerobic conditions without NADH by using HPLC. (*R*)- and (*S*)-DHD were used as the substrates, it showed that both (*R*)- and (*S*)-DHD can be transformed to DZN by K-07020. **(B)** The optimal pH for oxidase activity. Different lowercase letters indicate significant differences between groups (*P* < 0.05), except the groups between pH 5.5 and pH 7. * Represents the group of pH 5.5 was significantly different from the pH 7 group. **(C)** Kinetic analysis of oxidase activity. **(D)** The optimal temperature for oxidase activity. Oxidase activity analysis showed that the optimal conditions for K-07020 were a pH of 6.5 and a temperature of 41°C, and kinetic parameters for this reaction are Vmax of 52.2 μM/h and a Km of 4.2 μM.

In order to probe the DHD reverse transformation function of K-07020, DZNRs from *Lac* 20_92 and NATTS were selected as controls. The results of enzymatic assays indicated that all recombinant proteins showed DZNR activity, which is an ability to convert DZN to DHD (Supplementary Figure 3). But only K-07020 demonstrated oxidase activity, which is converting DHD to DZN. Moreover, both (*R*)-DHD and (*S*)-DHD could be converted into DZN by K-07020 and did not require additional coenzyme and anaerobic conditions (Figure 4A). Further oxidase activity analysis showed that the optimal conditions for K-07020 were a pH of 6.5 and a temperature of 41°C, and kinetic parameters for this reaction are *V*_*max*_ of 52.2 μM/h and a *K*_*m*_ of 4.2 μM. It was also observed that the optimal time for the enzyme–substrate reaction was 6 h at a DZN conversion rate of 57.3% when 80 μM DHD was added (Figures 4B–D).

### Sequence Analysis and Molecular Docking

The gene cluster for (*S*)-EQ-producing enzymes was next considered, as it is very similar for all reported (*S*)-EQ-producing bacteria, but not for genes similar to DDRCs, DHDRs and THDRs, as these have not been found in ZJ6. Moreover, 12-kb upstream and downstream of K-07020 no functional genes were identified (Supplementary Figure 5). Furthermore, K-07020 showed a conserved domain similarity to DZNRs (Supplementary Figure 6) that also have an OYE-like FMN binding domain (potential substrate binding or enzyme active site), a 4Fe–4S cluster motif or coenzyme binding motifs (potential enzyme active site).

**FIGURE 5:**
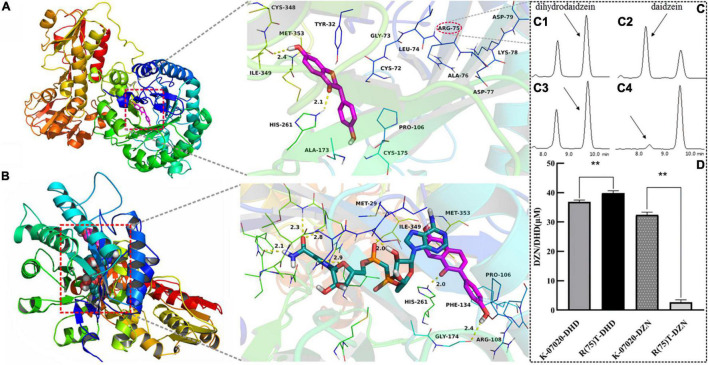
Analysis of oxidase key functional loci in K-07020. **(A)** DHD was docked into the binding site of K-07020. **(B)** DZN and NADH were docked into the binding site of K-07020. **(C)** Function detection of R(75)T K-07020 mutation by HPLC. **(C1,C2)** Respectively denote reductase and oxidase functions of K-07020; **(C3,C4)** respectively denote reductase and oxidase functions of R(75)T K-07020 mutation; **(D)** quantitative detection of K-07020 and R(75)T K-07020 mutation, DHD and DZN denote reductase and oxidase function detection, respectively, ** represents a *P*-value less than 0.01. The amino acid at position 75 (Arg) was identified as the key amino acid for DHD oxidase function in K-07020.

To further explore the new oxidase function of K-07020, a molecular docking study was used to screen this enzymatic activity site. DHD was docked into the binding site of K-07020 and the results are shown in Figure 5A. This study revealed that DHD was located to the hydrophobic site, surrounded by the residues Pro-106, Ala-173, Ile-349, and Met-353, forming a stable hydrophobic structure. Detailed analysis showed that the chromone scaffold of the DHD formed CH-π interactions with the residue Tyr-32 and Cys-72. Importantly, two key hydrogen bond interactions were observed between the DHD and the residues His-261 and Ile-349, which was the main interaction between the DHD and K-07020. For the reductase activity of K-07020, DZN and NADH were docked into the binding site of K-07020 (Figure 5B). The results showed that the residues Pro-106, Arg-108, Phe-134, Gly-174, His-261, Ile-349, and Met-353 may be the potential function cites of K-07020.

### Dihydrodaidzein Oxidase Function Site Assays

Following the results of molecular docking predication, seven potential functional regions in K-07020 were examined by site-directed mutagenesis with mutations consisting of several either consecutive or inconsecutive amino acid substitutions (Supplementary Table 1). In summary, six mutants that either showed no changes or significantly decreased both oxidase and reductase activities were obtained (Supplementary Table 4). However, one mutant with mutagenesis of the amino acid sequence at positions 72–74 and 79 (72CGLRADKD79/AKHRADKA), which significantly decreased DHD oxidase activity, was identified, but the DZN reductase activity was only slightly weakened. A further mutagenesis based on amino acid sequence at positions 75–78 (75RADK78/TTFI) revealed that the DHD oxidase activity of mutant 75–78 was almost lost, but there was no significant change in DZN reductase activity.

Point mutations at positions 75–78 were then further analyzed. The results from quantitative examination showed that the R(75)T mutant lost essentially all DHD oxidase function, but the corresponding DZN reductase activity was increased (*p* < 0.01). Finally, the amino acid at position 75 (Arg) was identified as the key amino acid for DHD oxidase function in K-07020 (Figures 5C,D). Moreover, site-directed mutagenesis of A(76)T, D(77)F, K(78)I, and D(79)A resulted in reduced DHD oxidase activity to varying degrees and no significant change in DZN reductase activity. Other point mutations at positions 72–74 were also assessed, and the enzyme activities of these mutants were either not significantly different or decreased to some extent for both DZN and DHD (Supplementary Table 4). After identifying the key active site of DHD oxidase function in K-07020, directed evolution of *Lac* 20_92 DZNR was performed by swapping in the active sites of K-07020. We found that a DZNR mutant with S(75)R resulted in DHD oxidase activity (Supplementary Figure 7).

### The Effect of (*S*)-EQ and Estradiol on Enzyme Activity of K-07020

**FIGURE 6:**
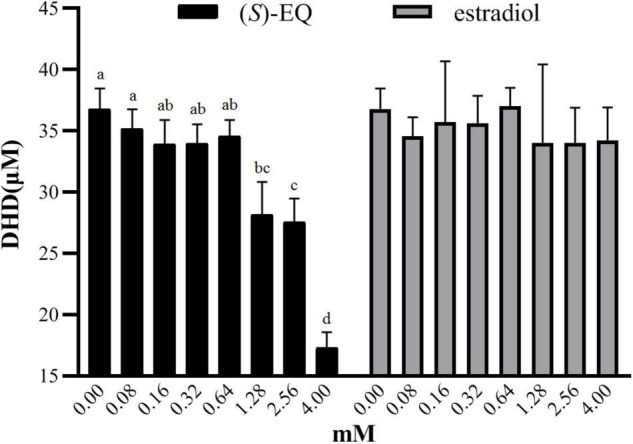
Effects of (*S*)-EQ and estradiol on the reductase and oxidase activity of K-07020. **(A)** Effects of (*S*)-EQ on DHD production when using DZN as reaction substrate. **(B)** Effects of estradiol on DHD production when using DZN as reaction substrate. Different lowercase letters indicate significant difference between groups (*P* < 0.05). With a concentration of (*S*)-EQ exceeding 1.28 mM, the reaction proceeded toward DZN formation and showed a concentration dependent increase in oxidase activity with increasing (*S*)-EQ concentrations.

(*S*)-EQ and its structurally similar compound estradiol were next applied to evaluate their effects on the enzyme activity of K-07020. When the concentration of the fermented DZN was 80 μM, the addition of (*S*)-EQ at concentrations ranging from 80 to 640 μM had no effect on enzyme activity (*p* > 0.05). However, with a concentration of (*S*)-EQ exceeding 1.28 mM, the reaction proceeded toward DZN formation and showed a concentration dependent increase in oxidase activity with increasing (*S*)-EQ concentrations (*p* < 0.05). In contrast, even when estradiol was added to a saturating concentration (4 mM), there were no significant effects (Figure 6). Moreover, fermentation of DHD was also evaluated with the addition of (*S*)-EQ or estradiol using the same method, and similar results were also observed.

### Metagenomics Analysis of K-07020

**FIGURE 7:**
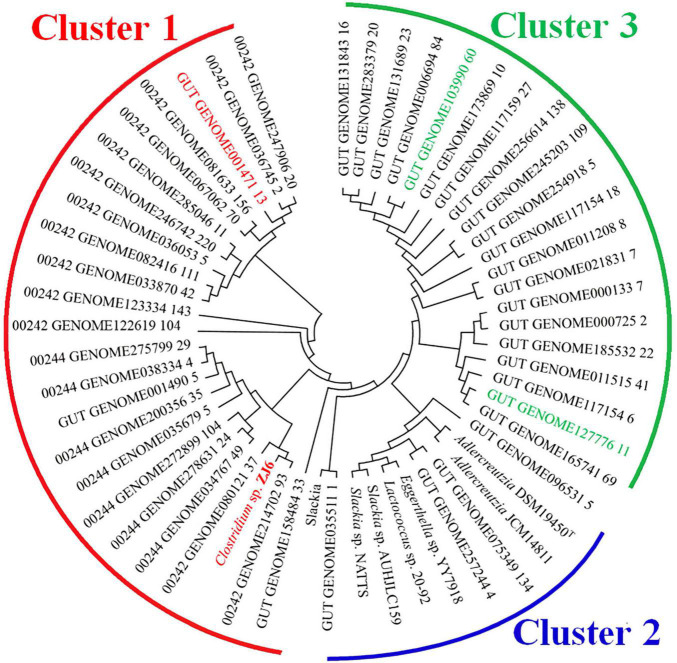
Bioinformatics analysis of K-07020 across the human gut metagenome. Cluster 1: the similarity of amino acid sequences with K-07020 were between 79–100%; Cluster 2: the similarity of amino acid sequences with K-07020 were between 44–46%; Cluster 3: the similarity of amino acid sequences with K-07020 were about 40%. Two genes showing low sequence similarity (marked by green) and one gene showing high sequence similarity (marked by red) with K-07020 were selected for functional identification *in vitro*.

K-07020 was identified as a new DZNR with both DZN reductase and DHD oxidase activities. To further explore the biological significance, K-07020 was used for amino acid sequence alignment analysis against the NCBI human and chicken gut metagenome database. A protein (GenBank: OUQ08445) with completely similarity to K-07020 was found in the chicken gut metagenome. In particular, 29 genes showed high similarity at the amino acid sequence level with K-07020, of which, the three reference strains MGYG-HGUT-00242 (containing 880 genomes), MGYG-HGUT-00244 (containing 692 genomes), and MGYG-HGUT-02689 (containing 33 genomes), had coverage greater than 80% for K-07020. Genes in the three reference strains were then used to blast K-07020 again and another twenty sequences were obtained. Following evolutionary genetic analysis, three clusters with K-07020 could be distributed in an evolutionary tree (Figure 7). Twenty-three genes showed a high similarity with K-07020 in the range from 79–100% and are listed in cluster 1, and almost half of these genes shared more than 90% similarity, particularly, 00242_GENOME214702_93, which was completely consistent with K-07020. In addition, cluster 2 was shown to have similarity with K-07020 between 44–46%, and most of these genes have been identified and reported as DZNRs.

**FIGURE 8:**
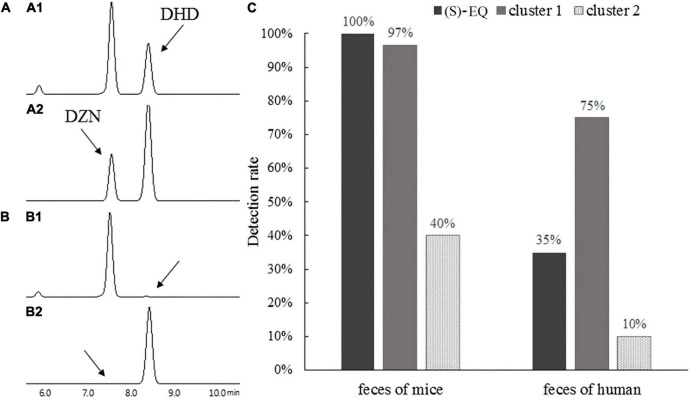
Detection of (*S*)-EQ and K-07020-like genes in human and mice feces. **(A1,A2)** Denote DZN reductase and DHD oxidase function from 00242_GENOMEO01471_13, showing that the genes in cluster 1 had DZN reductase and DHD oxidase activity. **(B1,B2)** Denote DZN reductase and DHD oxidase function from GUT_GENOME103990_60 and GUT_GENOME127776_11, however, no DZNRs were found in cluster 3. **(C)** Detection rate of (*S*)-EQ (HPLC) and K-07020-like genes (PCR) in human and mice feces.

In order to validate these predictions, two genes that showed about 40% sequence similarity in cluster 3 and one gene that showed 80% sequence similarity from cluster 1 with K-07020 were selected for functional identification *in vitro*. As expected from bioinformatics analysis, the genes in cluster 1 had DZN reductase and DHD oxidase activity (Figure 8A). However, no DZNR activity was found in these cluster 3 homologous genes (Figure 8B). To further investigate the distribution of K-07020-like genes, (*S*)-EQ production and PCR were used to detect the fecal samples of humans and mice. In mouse samples, the detection rates of both (*S*)-EQ production and K-07020-like genes were almost 100%. In human fecal samples, although the equol production rate was about 35%, the PCR positive ratio of K-07020-like genes was up to 75% (Figure 8C).

## Discussion

The strain ZJ6 was isolated from chick gut. In anaerobic fermentation, DZN was metabolized to DHD and (*S*)-EQ by this strain, to our surprise, when DHD was used as the substrate, DZN was also detected in the fermentation products. It is well known that DHDR and THDR can catalyze a reversible reaction, but not DZNR (Li, 2019). Therefore, this is the first discovery of the ability of (*S*)-EQ-producing bacteria to produce DZN using DHD. To determine the new characteristics of (*S*)-EQ production in ZJ6, whole genomes were sequenced, and (*S*)-EQ-producing genes were identified by Blast comparison with reported functional genes. A gene named K-07020 that showed 44.8% amino acid similarity with *Lac* 20_92 DZNR was obtained from bioinformatic analysis. Initially, the DZNR activity of K-07020 was identified in whole-cell extracts using an *E. coli* expression system. In addition, another soy isoflavone, genistein, was also detected in this experiment, and this showed that genistein could be converted to dihydrogenistein (Supplementary Figure 8). Presumably, K-07020 should be involved in the first step from DZN to (*S*)-EQ or genistein to 5-hydroxyequol. Otherwise, there were no other genes with similarity to DDRC, DHDR and THDR that were found in ZJ6. It has been reported that all of the major known genes for (*S*)-EQ production are located in a gene cluster with adjacent positions (Li, 2019), and for our analyses of potential gene clusters in ZJ6, 30 genes located up and downstream of K-07020 were investigated and some potential functional genes were also selected for enzyme activity detection *in vitro*, but no targets were found (Supplementary Figure 5). Therefore, ZJ6 may adopt a different mechanism to produce (*S*)-EQ and other functional genes need to be further explored.

K-07020 recombinant protein was purified and used for further functional assays *in vitro* in this work. A comparison of the DZN reductase activity between K-07020 and DZNRs from *Lac* 20_92 and NATTS showed that all of these recombinant proteins possessed reductase activity for the conversion of DZN to DHD, and these enzyme reaction systems require NAD(P)H and strictly anaerobic conditions (Shimada et al., 2010). Compared to *Lac* 20_92, K-07020 was highly specific for using NADH and not NADPH. Remarkably, DZN could be converted to *(R)*-DHD using the known DZNRs, while only (*S*)-DHD can be metabolized to (*S*)-EQ. DDRC was the key enzyme to affect the conversion of chiral molecules between *(R)*-DHD and (*S*)-DHD. However, about 70% of the product of (*S*)-DHD was detected in our K-07020 enzyme reactions (Figure 3A), meaning that K-07020 has the activity of directly producing (*S*)-DHD without DDRC.

Interestingly, only K-07020 was confirmed to have DHD oxidase activity that could reversibly catalyze R/S-DHD to DZN by dehydrogenation. This illustrated that K-07020 was a new bifunctional gene involved in producing (*S*)-EQ. Moreover, the DHD reaction kinetic revealed that the enzyme reaction rate of K-07020 was significantly lower than the rates of DZNRs, which could be relevant for its reversible enzyme activity. We also found that the optimal pH for DZN reductase activity (pH 7.5, Supplementary Figure 9) and DHD oxidase activity (pH 6.5) was different, and the direction of these reactions may be associated with the relative pH.

In our previous work, based on the four functional genes (*DZNR, DDRC, DHDR*, and *THDR*) from *Lac* 20_92, an engineering bacterium DDDT was successfully constructed and produced (*S*)-EQ *in vitro*. To further evaluate the role of K-07020, this gene was used to replace DZNR in the DDDT strain and the production of (*S*)-EQ was compared by fermentation experiments. We found that K-07020DDT exhibited higher (*S*)-EQ production than DDDT (*p* < 0.05), although the fermenting time was longer, which corresponds to the enzyme kinetics of K-07020 (Figure 3B). Moreover, DZN and (*S*)-EQ were found in the fermentation of K-07020DDT using DHD as a substrate. To be active as a DZN reductase, NADH and anaerobic conditions are essential. However, K-07020 still retained DHD oxidase activity without NADH under aerobic conditions, although it could not transform DHD to DZN completely *in vitro* (Figure 4).

We also investigated the influence of (*S*)-EQ and estradiol on the feedback enzyme activity of K-07020, and our results indicated that (*S*)-EQ could apparently promote DHD oxidase and inhibit DZN reductase activity at concentrations exceeding 1.28 mM. Although (*S*)-EQ is an estrogen-like chemical, estradiol did not exert similar effects in this experiment (Figure 6). These observations lead to the direct modulation of enzyme activity of K-07020 by (*S*)-EQ. Thus, a high concentration of (*S*)-EQ may be a feedback negative regulator of (*S*)-EQ-producing pathway in ZJ6.

Bioinformatics analysis indicated that both K-07020 and DZNRs have three conserved domains, an OYE-like flavin mononucleotide (FMN) binding domain, a 4Fe–4S cluster motif and a coenzyme binding motif (Kawada et al., 2018). Hydride transfer from cofactors plays an important role in many redox reactions, and often involves cofactors such as flavin, deazaflavin and nicotinamide adenine dinucleotide (NAD) (Kawada et al., 2018). Different reductases may have different coenzyme-binding sites involved in electron transfer, in which x amino acids from these three conserved domains may determine the selectivity of cofactors (Supplementary Figure 6). For the DZN reductase activity of K-07020, NADH but not NADPH provided hydrogen ions in DZN reduction reactions.

To investigate the new function of K-07020, molecular docking and site-directed mutagenesis was performed and used for the evaluation of the binding sites in K-07020 and DZN/DHD. We found that the residue Cys-72 was predicted to interact with the chromone scaffold of the DHD, but it had no effect on any enzyme activity. In contrast, neighboring residues, with Arg-75, were the key residues for the DHD oxidases activity of K-07020 (Figure 5). Moreover, we found that *Lac* 20_92 DZNR gained DHD oxidase activity when Ser-75 was mutated to Arg-75 (Supplementary Figure 7), which meant that Arg-75 may have been the key residue for DHD oxidase activity in DZNRs.

(*S*)-EQ-producing bacteria are necessary for producing (*S*)-EQ in humans and animals. However, many individuals who have these bacteria in their feces had undetectable levels of (*S*)-EQ, even after supplementation with a soy diet (Vazquez et al., 2017b; Iino et al., 2019; Yoshikata et al., 2019). Based on the (*S*)-EQ-producing features and the discovery of a bifunctional gene in ZJ6, metagenomics analysis and PCR detection were used to evaluate the distribution of K-07020-like genes in human and mouse intestines, respectively. We found more than 20 genes whose amino acid sequence similarity was higher than 80% to K-07020 from human gut metagenomic datasets (Figure 7). To our surprise, one gene and K-07020 were completely identical, and another gene showing 80% similarity to K-07020 was shown to have the same function as K-07020 (Figure 8A). Moreover, all genes in cluster 1 showed the same amino acid sequences in their functional sites as K-07020, which indicated that these genes may have the same enzyme activity (Figure 8B).

It has been reported that all rodents, but not humans, have the ability of producing (*S*)-EQ (Mayo et al., 2019). In this study, only part of the genes in cluster 2 were detected in mice. However, almost all of them were able to detect the production of (*S*)-EQ as well as the presence of cluster 1 genes. In humans, although the proportion of (*S*)-EQ producers were very low (∼35%), the detection rates for K-07020-like genes were high (∼75%). The positive ratio of these equol-producing genes showed no obvious correlation with (*S*)-EQ yield (Figure 8C).

Overall, it could be deduced that ZJ6-like bacteria or genes with (*S*)-EQ production roles may be commonly present in the intestine relative to known (*S*)-EQ-producing bacteria. (*S*)-EQ production capacity of an individual may not be dependent on the amount and species present in the gut, but rather tightly dependent on these colonies in a favorable intestinal environment. In these colonies, the bifunctional genes that are involved in (*S*)-EQ production are important targets for the metabolic regulation of (*S*)-EQ.

## Structural Formulas With Abbreviations for All Mentioned Compounds

Daidzein (DZN): 7-hydroxy-3-(4-hydroxyphenyl)-4H-chromen-4-one, C_15_H_10_O_4_.

*R/S*-dihydrodaidzein (*R/S*-DHD): (*R/S*)-2,3-Dihydro-7-hydroxy-3-(4-hydroxyphenyl)-4H-1-benzopyran-4-one, C_15_H_12_O_4_.

(*S*)-equol (EQ): (*S*)-3,4-Dihydro-3-(4-Hydroxyphenyl)-2H-1-benzopyran-7-ol, C_15_H_14_O_3_.

Genistein: 5,7-Dihydroxy-3-(4-Hydroxyphenyl)-4H-1-benzopyran-4-one, C_15_H_10_O_5_.

Dihydrogenistein: 2,3-Dihydro-5,7-dihydroxy-3-(4-hydroxyphenyl)-4H-1-benzopyran-4-one, C_15_H_12_O_5_.

## Data Availability Statement

The datasets presented in this study can be found in online repositories. The names of the repository/repositories and accession number(s) can be found below: https://www.ncbi.nlm.nih.gov/genbank/, AB558141.1; https://www.ncbi.nlm.nih.gov/genbank/, AP013105.1; https://www.ncbi.nlm.nih.gov/genbank/, JQ358709.1; https://www.ncbi.nlm.nih.gov/genbank/, AB646272.1; https://www.ncbi.nlm.nih.gov/genbank/, AP012211.1; https://www.ncbi.nlm.nih.gov/genbank/, QUN14249.1; https://www.ncbi.nlm.nih.gov/genbank/, BAJ22678; https://www.ncbi.nlm.nih.gov/genbank/, BAL46930; https://www.ncbi.nlm.nih.gov/genbank/, CP073631; and https://www.ncbi.nlm.nih.gov/genbank/, OUQ08445.

## Author Contributions

YH and YY designed the experiment, performed the (*S*)-EQ-production and functional genetic screening experiments, and wrote the manuscript with input from all authors who reviewed the final manuscript. YH, CY, and CS performed the enzyme activity analysis and site-directed mutagenesis experiments. YH, BL, LC, CZ, and HC analyzed the data. YH, WZ, and YY performed the metagenomics analysis and sequence diversity analysis. All authors contributed to the article and approved the submitted version.

## Conflict of Interest

The authors declare that the research was conducted in the absence of any commercial or financial relationships that could be construed as a potential conflict of interest.

## Publisher’s Note

All claims expressed in this article are solely those of the authors and do not necessarily represent those of their affiliated organizations, or those of the publisher, the editors and the reviewers. Any product that may be evaluated in this article, or claim that may be made by its manufacturer, is not guaranteed or endorsed by the publisher.

## Footnotes

1
https://blast.ncbi.nlm.nih.gov/Blast.cgi

2
https://www.ncbi.nlm.nih.gov/Structure/cdd/wrpsb.cgi

3
https://myhits.sib.swiss/cgi-bin/motif_scan

4
www.pymol.org

5
http://smart.embl-heidelberg.de/
