# Supplementary material for: Characterization and Identification of a New Daidzein Reductase Involved in (S)-Equol Biosynthesis in Clostridium sp. ZJ6
Source: Front Microbiol. 2022 May 20;13:901745. doi: 10.3389/fmicb.2022.901745 (PMC9164157; doi:10.3389/fmicb.2022.901745)
Supplement: Supplementary file 1 [file Table_1.docx]

Supplementary Material

# Supplementary Tables

**Supplementary** **Table S1.** Primers used for region mutations of K-07020

| Mutants | Primer | Nucleotide sequence |
| --- | --- | --- |
| 105 FTKTF 109 | fp1 | agcagccatcaccatcatcaccacagccagatgaaaaacaaatattaccctca |
|  | rp1_1 | tgctgcaaatgtcttagtaaatgccaattgtagaccaggagttgctccatgttct |
|  | fp2_1 | tacaattggcatttactaagacatttgcagcatttgtaggaagtgctgatgt |
|  | rp2 | agcattatgcggccgcaagcttgtcgaccttagatttgtctggctgctataaatc |
| 72 AKHRADKA 79 | rp1_2 | aaattcagctttgtccgctctatgcttagcagatgtcataggaacaggtacag |
|  | fp2_2 | gtacctgttcctatgacatctgctaagcatagagcggacaaagctgaattt |
| 75 TTFI 78 | rp1_3 | aaattcatctatgaacgttgttaaaccacaagatgtcataggaacaggtacag |
|  | fp2_3 | gtacctgttcctatgacatcttgtggtttaacaacgttcatagatgaattt |
| 259 KTLDTD 264 | rp1_4 | tcgagagtcagtgtcaagtgtcttacccatcatatttaaatgagatacaccttct |
|  | fp2_4 | atatgatgggtaagacacttgacactgactctcgacaattcttattgccaa |
| 347 DATDAGT 353 | rp1_5 | ctaacgttcctgcatcagtagcatcgatcatacaattgatacatggtttaatatc |
|  | fp2_5 | tatgatcgatgctactgatgcaggaacgttaggtcatacaccaatccattgtac |
| 29 KDAAQ 33 | rp1_6 | tgtctatcagtgactgtaccatctggattaccttgggccgcatccttagaatt |
|  | fp2_6 | aattctaaggatgcggcccaaggtaatccagatggtacagtcactgatagaca |
| 173 TED 175 | rp1_7 | gtgataagaaatttgtaggaatatcttcagtagccccatgaatttcaaggac |
|  | fp2_7 | attcatggggctactgaagatattcctacaaatttcttatcacctcatgataac |
| 133 KY 134 | rp1_8 | agctctaggcattttatacttcatttcataccatggttcaaaagtaattggtg |
|  | fp2_8 | ccatggtatgaaatgaagtataaaatgcctagagctttaactattgaagaaattca |

Note: mutated amino acids or nucleotides are underlined, and numbers indicate amino acid positions in K-07020. All the primers for fp1 and rp2 were 105 FTKTF 109 fp1 and 105 FTKTF 109 rp2.

**Supplementary** **Table S2.** Primers used for point mutations of K-07020

| Mutants | Primer | Nucleotide sequence |
| --- | --- | --- |
| P106T | fp1 | agcagccatcaccatcatcaccacagccagatgaaaaacaaatattaccctca |
|  | rp1_9 | tgctgcatctctaccagtatgtgccaattgtagaccaggagttgctccatgttct |
|  | fp2_9 | tacaattggcacatactggtagagatgcagcatttgtaggaagtgctgatgt |
|  | rp2 | agcattatgcggccgcaagcttgtcgaccttagatttgtctggctgctataaatc |
| R108T | rp1_10 | tgctgcatctgtaccaggatgtgccaattgtagaccaggagttgctccatgttct |
|  | fp2_10 | tacaattggcacatcctggtacagatgcagcatttgtaggaagtgctgatgt |
| C72V | rp1_11 | tcatctttgtccgctcttaaaccaacagatgtcataggaacaggtacagcat |
|  | fp2_11 | cctatgacatctgttggtttaagagcggacaaagatgaatttattgctgga |
| G73K | rp1_12 | aaattcatctttgtccgctcttaacttacaagatgtcataggaacaggtacag |
|  | fp2_12 | gtacctgttcctatgacatcttgtaagttaagagcggacaaagatgaattt |
| L74H | rp1_13 | aaattcatctttgtccgctctatgaccacaagatgtcataggaacaggtacag |
|  | fp2_13 | gtacctgttcctatgacatcttgtggtcatagagcggacaaagatgaattt |
| R75T | rp1_14 | aaattcatctttgtccgctgttaaaccacaagatgtcataggaacaggtacag |
|  | fp2_14 | gtacctgttcctatgacatcttgtggtttaacagcggacaaagatgaattt |
| A76T | rp1_15 | aaattcatctttgtccgttcttaaaccacaagatgtcataggaacaggtacag |
|  | fp2_15 | gtacctgttcctatgacatcttgtggtttaagaacggacaaagatgaattt |
| D77F | rp1_16 | aaattcatctttgaacgctcttaaaccacaagatgtcataggaacaggtacag |
|  | fp2_16 | gtacctgttcctatgacatcttgtggtttaagagcgttcaaagatgaattt |
| K78I | rp1_17 | aaattcatctatgtccgctcttaaaccacaagatgtcataggaacaggtacag |
|  | fp2_17 | gtacctgttcctatgacatcttgtggtttaagagcggacatagatgaattt |
| D79A | rp1_18 | aaattcagctttgtccgctcttaaaccacaagatgtcataggaacaggtacag |
|  | fp2_18 | gtacctgttcctatgacatcttgtggtttaagagcggacaaagctgaattt |
| H261L | rp1_19 | tcgagatgcagttgcaagtgtcccacccatcatatttaaatgagatacaccttct |
|  | fp2_19 | atatgatgggtgggacacttgcaactgcatctcgacaattcttattgccaa |
| A262D | rp1_20 | tcgagatgcagtgtcatgtgtcccacccatcatatttaaatgagatacaccttct |
|  | fp2_20 | atatgatgggtgggacacatgacactgcatctcgacaattcttattgccaa |
| I349H | rp1_21 | tggattggtgtatgacctaacattcctttatcatgacaaccgatcatacaattga |
|  | fp2_21 | gatcggttgtcatgataaaggaatgttaggtcatacaccaatccattgtac |
| Y32A | rp1_22 | tgtctatcagtgactgtaccatctggattacctaaggccgttcccatagaatt |
|  | fp2_22 | aattctatgggaacggccttaggtaatccagatggtacagtcactgatagaca |
| A173C | rp1_23 | gtgataagaaatttgtaggaatacatccacaagccccatgaatttcaaggac |
|  | fp2_23 | catggggcttgtggatgtattcctacaaatttcttatcacctcatgataac |
| G174E | rp1_24 | gtgataagaaatttgtaggaatacattcagcagccccatgaatttcaaggac |
|  | fp2_24 | attcatggggctgctgaatgtattcctacaaatttcttatcacctcatgataac |

Note: mutated nucleotides are underlined, and numbers in parentheses indicate amino acid positions in K-07020. All the primers for fp1 and rp2 were P106T fp1 and P106T rp2.

**Supplementary** **Table S3.** Primers used for site-directed mutations of Lac 20-92 DZNR

| Mutants | Primer | Nucleotide sequence |
| --- | --- | --- |
| 72 CGLRADK 78 | fp3 | agcagccatcaccatcatcaccacagccagatgaagaacaagttctatccga |
|  | rp3_1 | gatgtagttgtctttgtccgcgcgcagtccgcaatggtggaactgggtcacg |
|  | fp4_1 | gtgacccagttccaccattgcggactgcgcgcggacaaagacaactacatcg |
|  | rp4 | agcattatgcggccgcaagcttgtcgaccctacaggttgcagccagcgatgt |
| S75R | rp3_2 | gatgtagttgtcgctggccaggcgcagtccgacatggtggaactgggtcacg |
|  | fp4_2 | gtgacccagttccaccatgtcggactgcgcctggccagcgacaactacatcg |

Note: mutated amino acid or nucleotides are underlined, numbers in parentheses indicate amino acid positions in *Lac* 20-92 DZNR. All the primers of fp3 and rp4 were identical.

**Supplementary** **Table S4.** Enzyme activity detection of K-07020 mutants

| Mutants | DZN reductase | DHD oxidases |
| --- | --- | --- |
| 105 FTKTF 109 | lost | lost |
| P106T | no significant change | no significant change |
| R108T | no significant change | no significant change |
| 72 AKHRADKA 79 | slightly weakened | significantly weakened |
| C72V | no significant change | no significant change |
| G73K | no significant change | no significant change |
| L74H | no significant change | no significant change |
| D79A | no significant change | significantly weakened |
| 75 TTFI 78 | no significant change | almost lost |
| R75T | slightly increased | almost lost |
| A76T | no significant change | significantly weakened |
| D77F | no significant change | significantly weakened |
| K78I | no significant change | significantly weakened |
| 259 KTLDTD 264 | lost | lost |
| H261L | no significant change | no significant change |
| A262D | no significant change | no significant change |
| 347 DATDAGT 353 | significantly weakened | significantly weakened |
| I349H | no significant change | no significant change |
| 29 KDAAQ 33 | lost | lost |
| Y32A | no significant change | no significant change |
| 173 TED 175 | significantly weakened | significantly weakened |
| A173C | no significant change | no significant change |
| G174E | no significant change | significantly weakened |
| 133 KY 134 | no significant change | no significant change |

Note: mutated amino acids are underlined, and numbers in parentheses indicate amino acid positions in K-07020. Six mutants were not the key residues for the oxidase activity of K07020, and these mutants either showed no change, were lost, or significantly decreased all enzyme activities. However, one mutant (72 AKHRADKA 79) was identified as significantly weakening DHD oxidase activity and also weakening DZN reductase activity to some extent. A further mutagenesis based on 75 (Arg) was identified as the key amino acid for DHD oxidase function in K-07020.

# Supplementary Figures

##
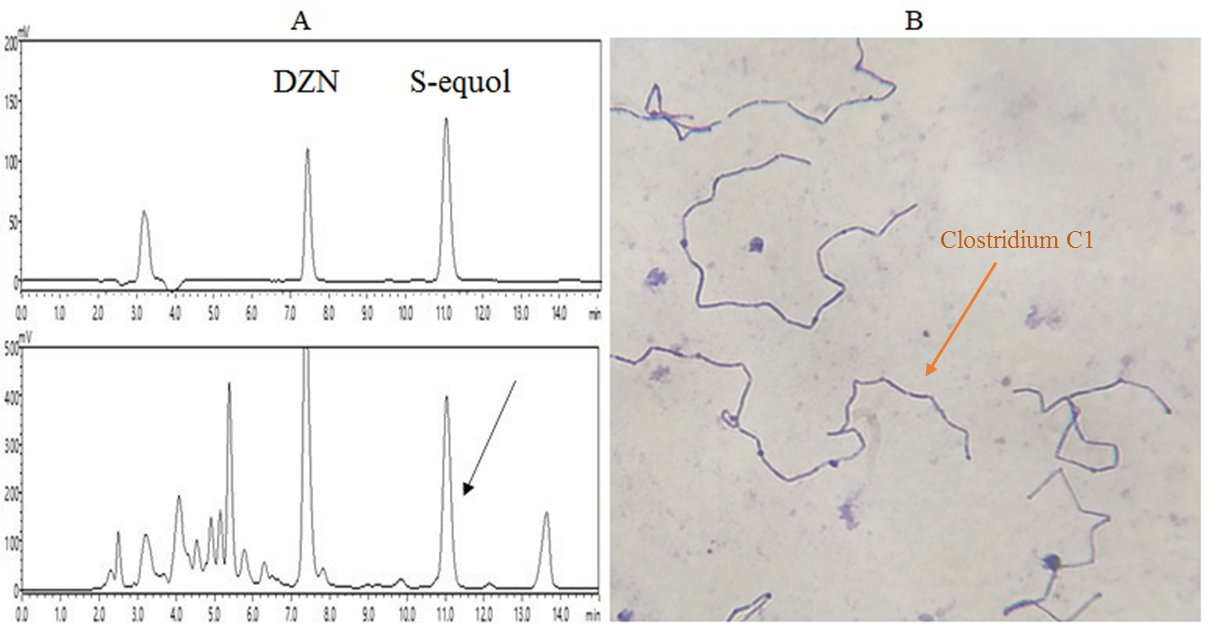
Supplementary Figure S1. Characteristics of *Clostridium* sp. ZJ6 for S-equol production

A: HPLC results from DZN fermentation by *Clostridium* sp. ZJ6; B: gram staining of *Clostridium* sp. ZJ6.


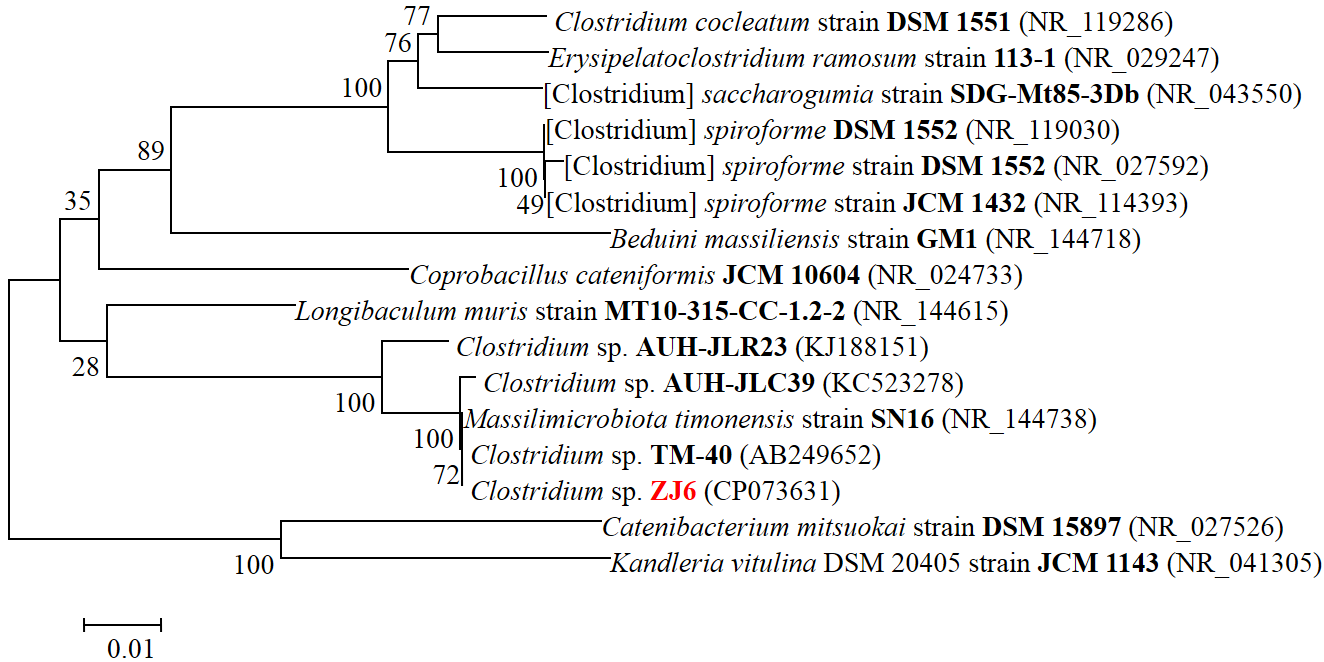


## Supplementary Figure S2. Phylogenetic tree analysis of 16S rRNA genes from *Clostridium* sp. ZJ6

Accession numbers are shown in parenthesis. Numbers at the branch points indicate the bootstrap values, and the scale bar corresponds to 0.01 substitutions per nucleotide position; the name of *Clostridium* sp. ZJ6 in Genebank was *Clostridium* sp. C1.


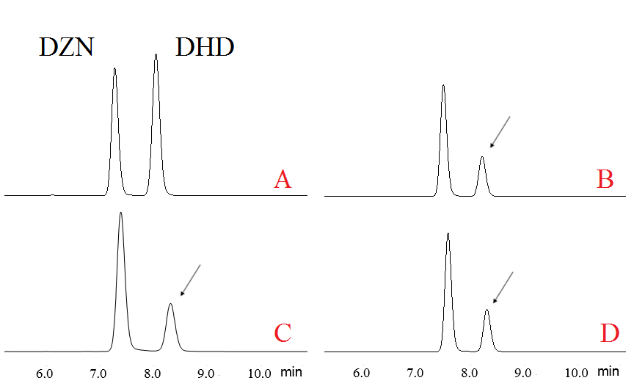


## Supplementary Figure S3. Reductase activity detection of K-07020, *Lac* 20_92 DZNR and NATTS DZNR

A: standards; B: activity detection of K-07020 recombinant protein; C: activity detection of *Lac* 20_92 DZNR recombinant protein; D: activity detection of NATTS DZNR recombinant protein.


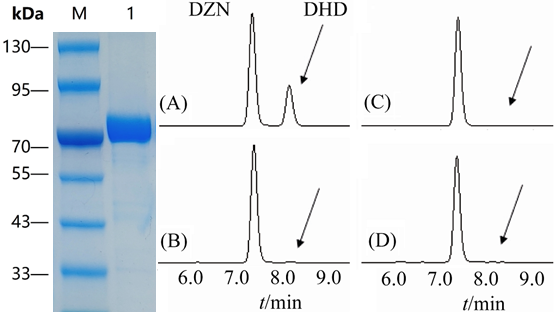


## Supplementary Figure S4. Reductase activity assay of K-07020

M: protein marker; 1: purified recombinant protein from K-07020. A: activity detection of K-07020 recombinant protein in NADH and anaerobic condition. B: activity detection of K-07020 recombinant protein in NADPH and anaerobic condition. C: activity detection of K-07020 recombinant protein in NADH and aerobic condition. D: activity detection without K-07020 recombinant protein. This reveals that recombinant proteins from K-07020 possessed reductase

activity for conversion of DZN to DHD, and the enzyme reaction system requires NADH, but not NADPH and strictly anaerobic conditions.


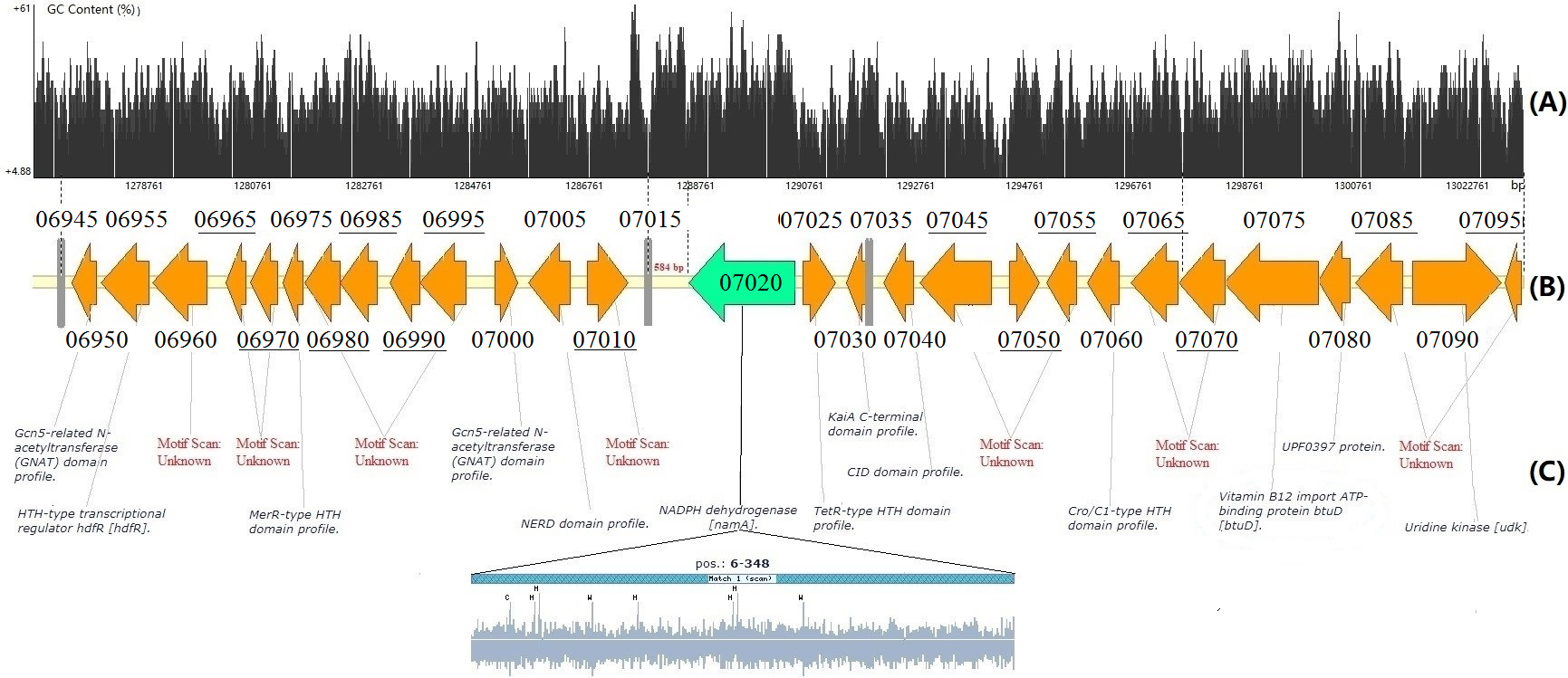


## Supplementary Figure S5. Analysis of upstream and downstream genes of K-07020 in *Clostridium* sp. ZJ6

A: GC content. B: sequence information of upstream and downstream genes of K-07020 predicted by whole genome sequencing. C: functional prediction analysis of each CDS. K-07020 was predicted as a NADPH dehydrogenase, but no other (*S*)-equol-related genes were found. The genes with unknown functions were also select for enzyme activity detection *in vitro*, but no target was found.


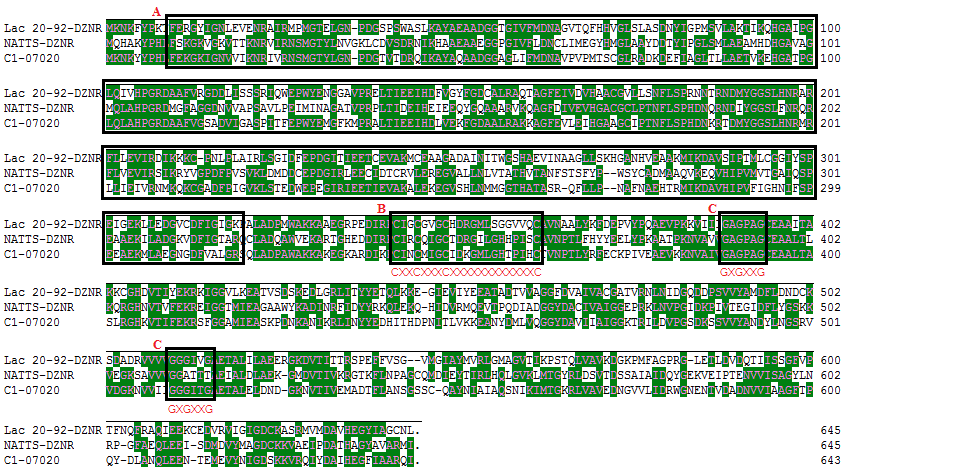


## Supplementary Figure S6. Conserved domain analysis of K-07020

The same amino acid residues are represented in green. A: OYE-like FMN binding domain. B: 4Fe-4S Cluster Motif. C: coenzyme binding motifs. The common amino acid residues of the 4Fe-4S cluster motif and coenzyme binding motifs are below box B and C, respectively.


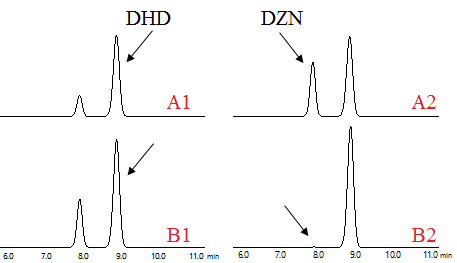


## Supplementary Figure S7. Detection of DHD oxidase activity in *Lac* 20_92 DZNR mutants

A1 and A2 denote DZN reductase and DHD oxidase function, respecitively, for the detection of mutants with S75R. the results showed that mutant S75R of *Lac* 20_92 DZNR had the same function as K-07020. B2 denotes DZN reductase and DHD oxidase function detection of mutants with 72 CGLRADK 78, revealing that this mutant still had no DHD oxidase function.


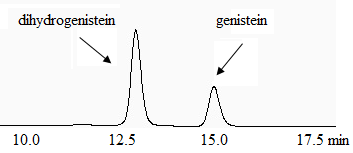


## Supplementary Figure S8. Genistein fermentation of K-07020

Recombinant protein of K-07020 could convert genistein to dihydrogenistein.


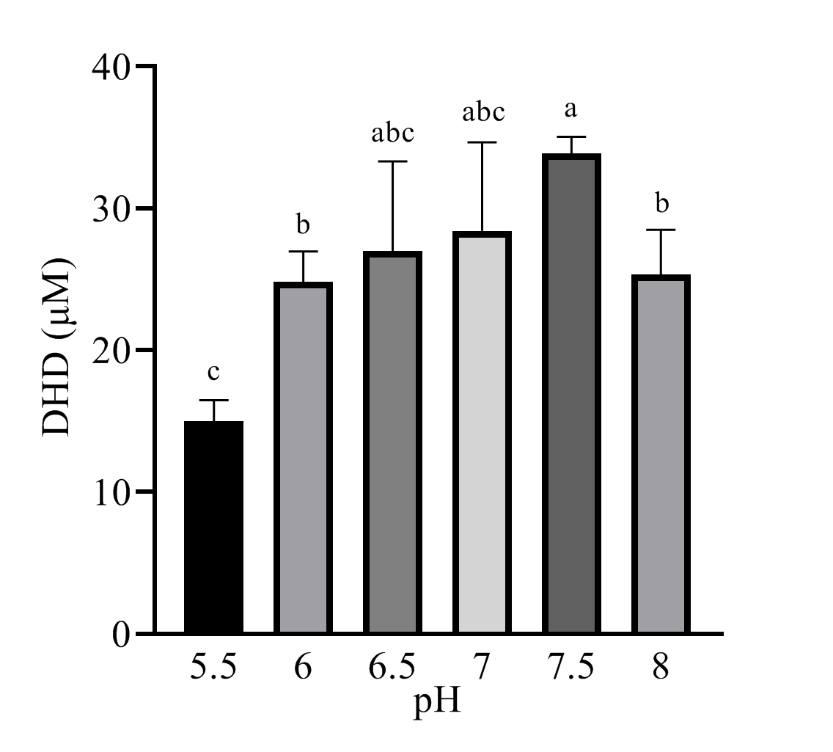


## Supplementary Figure S9. The optimal pH for DZN reductase of K-07020

*E. coli* of K-07020 were inducted and expressed in LB medium to an OD_600_ of 1.5 (~2 × 10^7^ cfu/ml), and 80-μM DZN was added to a whole-cell biocatalyst under anaerobic conditions. Following incubation at 37°C for 4 h, samples were used for HPLC detection. Different lowercase letters indicate significant differences between groups (P < 0.05).
